# CRISPR Technologies in Type 2 Diabetes: From Mechanistic Insights to Therapeutic Discovery

**DOI:** 10.3390/ijms27188360

**Published:** 2026-09-19

**Authors:** Shuangshuang Fan, Ruipu Liang, Teng Fei

**Affiliations:** 1 Interdisciplinary Research Center for Brain-Computer Interface, Key Laboratory of Research and Development of Liaoning Province, College of Life and Health Sciences, Northeastern University, Shenyang 110819, China; fanss_email@163.com (S.F.); liangruipu@163.com (R.L.); 2 National Frontiers Science Center for Industrial Intelligence and Systems Optimization, Northeastern University, Shenyang 110819, China; 3 Key Laboratory of Data Analytics and Optimization for Smart Industry, Northeastern University, Ministry of Education, Shenyang 110819, China

**Keywords:** diabetes, metabolism, CRISPR, glucose, lipid, insulin, gene therapy, drug target

## Abstract

Type 2 diabetes (T2D) is a complex metabolic disorder driven by the interplay of genetic susceptibility, *β*-cell dysfunction, and insulin resistance. Although genome-wide association studies (GWAS) have identified numerous T2D risk loci, translating these genetic findings into biological mechanisms and therapeutic strategies remains challenging. The emergence of CRISPR (Clustered Regularly Interspaced Short Palindromic Repeats)-based technologies has provided powerful tools for precise genome manipulation and functional interrogation of disease-associated genes and genetic variations. This review summarizes the development of CRISPR technologies and highlights their applications in T2D research. We review the application of CRISPR technologies in T2D genetic factor analysis and high-throughput phenotypic screening, with a focus on three key areas: glucose metabolism, lipid metabolism, and pancreatic functionality. We further examine the role of CRISPR in therapeutics that target pancreatic *β*-cells via gene correction, stress protection, and epigenetic reprogramming. Finally, we briefly discuss the major challenges encountered in the applications of CRISPR technology, including delivery efficiency, off-target effects, safety concerns and ethical considerations.

## 1. Introduction

Diabetes mellitus is a complex and chronic metabolic disorder whose global prevalence has sharply increased over the past few decades, currently affecting over 580 million adults. This number is expected to rise to nearly 850 million by 2050, with Type 2 diabetes (T2D) accounting for more than 90%, approximately 480 million individuals. In recent years, the disease has increasingly been diagnosed at younger ages, constituting one of the most significant public health challenges of this century [1]. The pathophysiology of diabetes extends beyond simple hyperglycemia, encompassing a multifactorial metabolic disorder driven by insulin resistance, progressive pancreatic *β*-cell dysfunction, and upregulated hepatic gluconeogenesis [2]. The mechanisms underlying its onset involve complex gene–environment interactions, including hundreds of genetic susceptibility loci identified by genome-wide association studies (GWAS), as well as significant interactions with environmental factors such as obesity, sedentary lifestyles, and nutritional excess [3]. Although current pharmacological treatments, such as metformin (which improves insulin sensitivity), sulfonylureas (which promote insulin secretion), and emerging glucagon-like peptide 1 receptor agonists (GLP-1 RAs) and sodium-glucose co-transporter 2 inhibitors (SGLT2i), have achieved apparent success in blood glucose control, some patients still experience disease progression and are at risk for devastating complications, including cardiovascular disease, nephropathy, neuropathy, and retinopathy [4,5,6,7,8,9,10]. These complications resulted in approximately 3.4 million deaths in 2024. This underscores the urgent need to fundamentally elucidate the molecular mechanisms of T2D and develop revolutionary therapeutic strategies [1].

In recent years, the emergence and development of Clustered Regularly Interspaced Short Palindromic Repeats (CRISPR) gene editing technology has brought a paradigm shift to biomedical research [11,12]. Derivative technologies, particularly CRISPR-Cas9-based gene knockout (KO), base editor, prime editor, and epigenome editors such as CRISPR interference (CRISPRi)/activation (CRISPRa), have enabled highly efficient perturbations and functional interrogation of target genes or genetic elements across the genome [13]. Compared to traditional RNA interference (RNAi)-based technologies, CRISPR offers higher editing efficiency, fewer off-target effects when properly designed, and more lasting genetic modifications, making it particularly suited for studying the complex regulatory networks of multi-gene diseases like T2D [14,15,16,17]. By constructing in vitro and in vivo models that closely resemble human diseases, CRISPR technology is revealing the etiology of T2D with unprecedented depth.

In the field of T2D research, the application of CRISPR technology is reflected in three key areas. First, humanized disease models can be constructed through gene editing, such as introducing specific T2D risk alleles into human pluripotent stem cells (hPSCs) and differentiating them into pancreatic *β*-cells or hepatocytes, allowing for gene function studies in a human genetic context [18,19,20]. Second, through genome-wide or high-throughput CRISPR screening, functional genes and pathways crucial to T2D-related phenotypes—such as *β*-cell survival, insulin signaling, and glucose-lipid metabolism—can be systematically identified, thereby dissecting the complex molecular mechanisms underlying T2D [19,20]. Third, CRISPR technology can be directly utilized as a therapeutic tool, for example, editing key genes to protect *β*-cell function and/or enhance insulin sensitivity, or identifying potential drug targets and mechanism-of-action through CRISPR screening, thus accelerating the development of precision medicine [19,21].

This review aims to explore in-depth how CRISPR technology is reshaping our understanding and treatment strategies for T2D. We review the evolution of CRISPR technologies and summarize the major CRISPR strategies applied (especially since 2023) in T2D-related research as well as therapeutics development. This work is intended as a narrative rather than a systematic review. Literature was searched in PubMed/MEDLINE, Web of Science, Scopus, and bioRxiv, using terms including CRISPR, type 2 diabetes, glucose, lipid, *β*-cell, insulin secretion, CRISPR screen, gene editing, base editing, prime editing, lipid nanoparticles, and therapeutic delivery. To make a fair and smooth review, several key works published before 2023 were also included.

## 2. The Evolution of CRISPR Technologies

CRISPR-Cas systems, first adapted as programmable genome editing tools in 2012 following the demonstration of RNA-guided DNA cleavage by Cas9, have rapidly evolved into a highly diversified molecular toolbox for genome engineering, transcriptional regulation, epigenome modulation, RNA targeting and functional genomics. This modular evolution has enabled the transformation of CRISPR from a nuclease-based editing system into a multifunctional platform for biomedical research [11,12,22,23,24,25].

### 2.1. CRISPR as an Evolving Genome Engineering Platform

**Figure 1:**
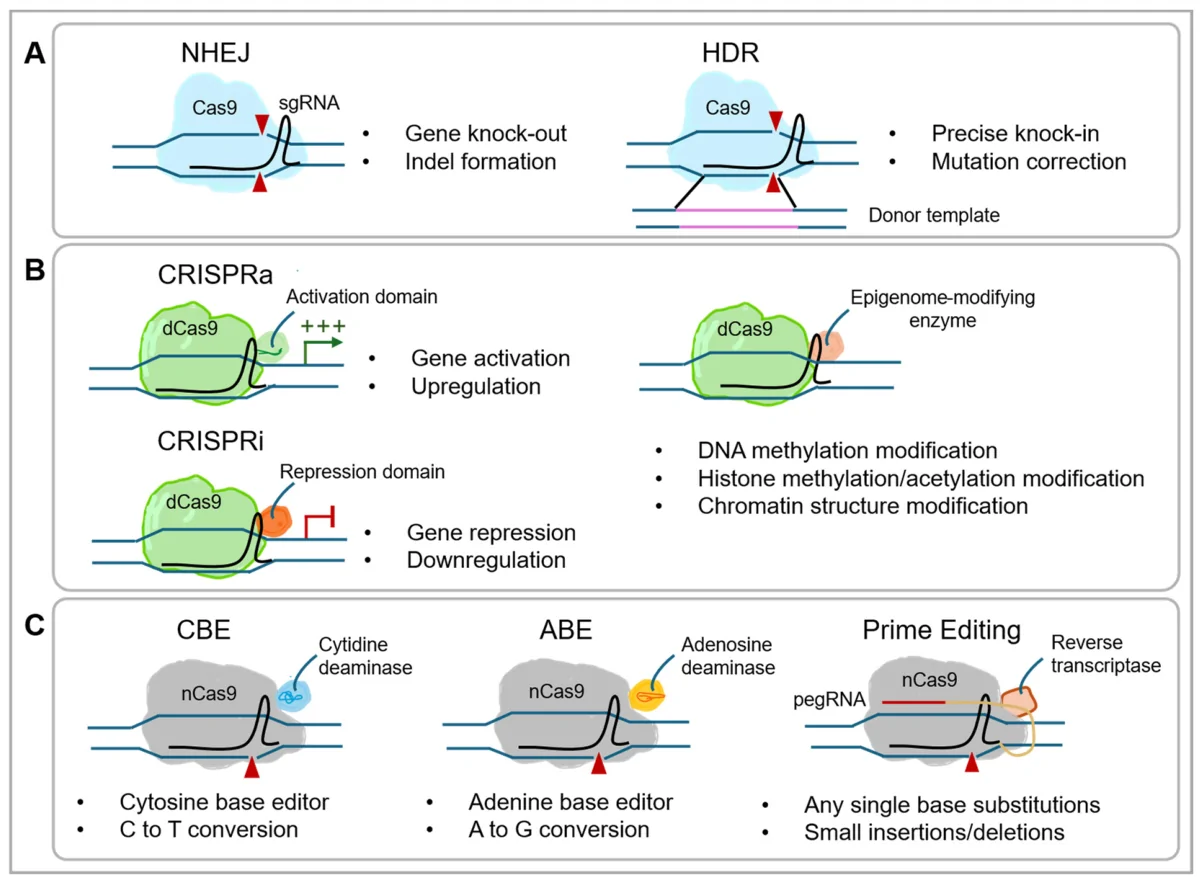
Overview of major CRISPR/Cas9-based genome engineering tools. (**A**) Cas9 nuclease induces sgRNA-guided double-strand DNA breaks at target sites, which can be repaired by endogenous DNA repair mechanisms to generate indels or targeted genomic modifications. (**B**) dCas9 retains its DNA-targeting capability while lacking nuclease activity and can be coupled with transcriptional activators, repressors, or specific epigenetic modifiers to control targeted gene expression. (**C**) nCas9, which introduces single-strand DNA breaks, can be fused or associated with different editing effectors to enable precise genome manipulation, including base editing and prime editing. These CRISPR-based approaches provide versatile tools for gene knockout, transcriptional perturbation, and precise genome editing. Red triangle: DNA break site.

The initial application of CRISPR technology was concentrated on *Streptococcus pyogenes* Cas9 (SpCas9)-mediated DNA cleavage, which generates DNA double-strand breaks (DSBs) repaired primarily via the non-homologous end joining (NHEJ) pathway, thereby generating indels and enabling efficient gene knockout for protein-coding genes. On the other hand, the DSB can also be repaired via the homology-directed repair (HDR) pathway in the presence of an appropriate DNA donor template, leading to a precise genome editing outcome [26] (Figure 1A). Subsequent repurposing of catalytically inactive Cas9 (dCas9) expanded its utility beyond genome editing to transcriptional regulation (CRISPRi/a) and epigenome engineering (CRISPR-EE). In these systems, dCas9 can be fused with transcriptional activators or repressors, as well as epigenetic modifiers such as TET1 and DNMT3A, enabling programmable control of DNA methylation states and transcriptional memo [27,28,29] (Figure 1B).

### 2.2. Precision Genome Editing and Beyond

Beyond simple gene knockout, CRISPR technologies have expanded into precise genome editing strategies that avoid DSBs. Base editing systems, including classic cytosine base editors (CBEs) and adenine base editors (ABEs), utilize Cas9 nickase (nCas9) fused with deaminase enzymes to achieve targeted C•G-to-T•A or A•T-to-G•C conversions, respectively, without inducing double-strand DNA cleavage [30,31]. These systems have been further extended by transversion base editors and dual-base editors [32,33,34], enabling broader mutational outcomes at single-nucleotide resolution. Prime editing represents a further advancement in precision genome engineering, coupling nCas9 to an engineered reverse transcriptase and using a prime editing guide RNA (pegRNA), thereby enabling all 12 possible base substitutions as well as small insertions and deletions without requiring DSBs or donor DNA templates [35] (Figure 1C).

In parallel, Cas12 family nucleases, particularly Cas12a, provide staggered DNA cleavage (cis-activity) and possesses collateral single-stranded DNA cleavage activity (trans-activity), which has been leveraged for multiplex genome editing and molecular diagnostic applications [36]. Recent discovery of compact CRISPR-associated systems, including Casj, Cas12l, and transposon-derived TnpB systems, has further expanded the engineering landscape by enabling smaller, more easily deliverable effectors suitable for in vivo applications [37,38,39]. In addition to DNA-level editing, CRISPR systems have been adapted for RNA targeting and transcriptome modulation. Cas13-based systems provide sequence-specific RNA cleavage, while exhibiting collateral RNase activity that enables applications in RNA knockdown, editing, imaging and molecular diagnostics [40,41,42,43]. The details and emerging CRISPR-related tools can be found in specified reviews [43,44,45].

### 2.3. Modified Backbones for CRISPR Technology

CRISPR-Cas activity is limited by nuclease-sensitive RNA backbones and insufficient editing specificity. Chemical gRNA backbone modification improves stability, activity, and specificity without sequence alteration. Phosphorothioate (PS) linkages are best characterized: terminal 2′-O-methyl/PS modifications enhance serum stability and nuclease resistance while maintaining or improving editing, and positional mapping defines PS tolerance across crRNA [46,47,48]. A recent bioRxiv preprint reported that combining site-specific PS with ribose replacement (2′-fluoro RNA, 2′-H DNA) restores Cas9 binding and high-fidelity editing in ribose-free crRNA, showing that PS compensates for 2′-OH [49]. PS-DNA oligos also modify Cas9, enabling efficient delivery of single and bi-specific Cas9/gRNA dimers with reduced off-target effects [50].

Peptide nucleic acids (PNAs) offer a specificity-oriented alternative. Through Watson–Crick pairing with gRNA, PNAs act as antisense regulators of Cas9. A 14-mer PNA targeting gRNA positions 7–20 (a PAM-distal region) raised Cas9 specificity up to 10-fold in on-target/off-target and allele-specific contexts [51]. PNAs thus function as programmable regulators that reshape gRNA conformation for high-fidelity recognition. Optimal future gRNAs should integrate PS-based stability, PNA-based specificity, and site-specific ribose substitutions for activity rather than relying on any single modification.

### 2.4. CRISPR-Based Functional Genomics Platforms and High-Throughput Screening

**Figure 2:**
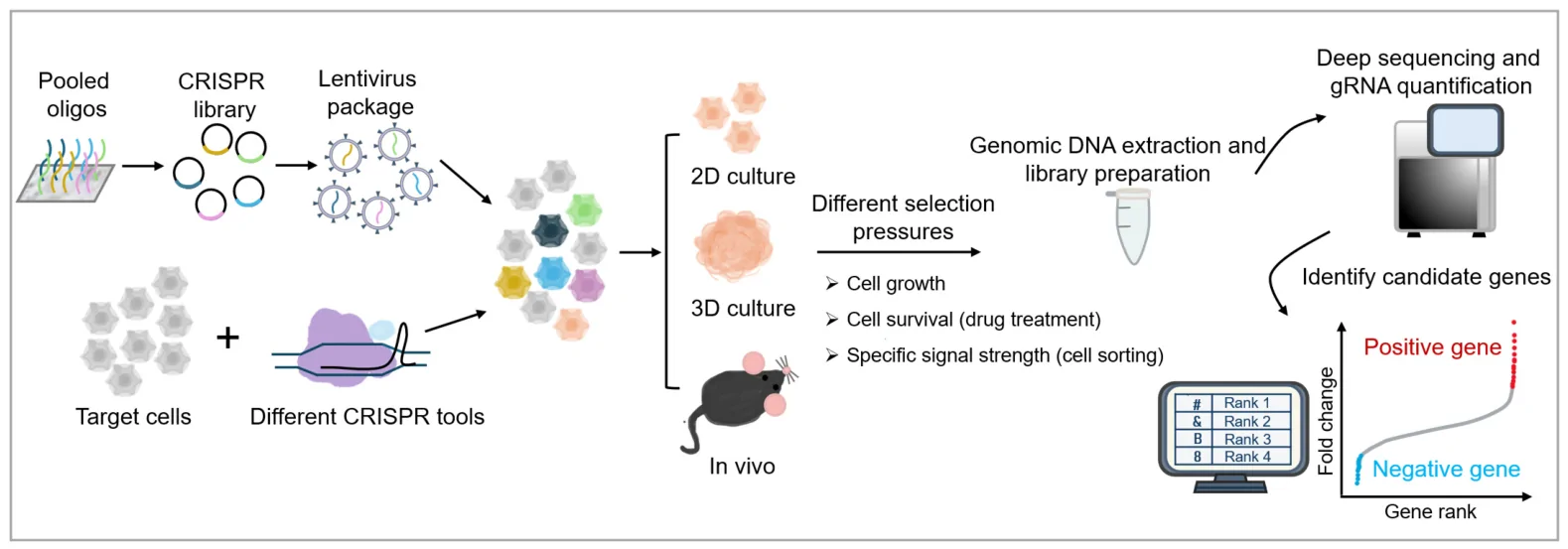
Schematic overview of representative pooled CRISPR screening workflow for functional genomic studies. A pooled CRISPR screening experiment generally involves the design and generation of a large-scale sgRNA library, followed by delivery into target cells and phenotypic selection. CRISPR libraries can be introduced into cellular or in vivo models to systematically perturb gene function. Edited cells are subsequently subjected to defined selection pressures, such as cell fitness, drug treatment or other disease-relevant conditions. Following selection, genomic DNA is extracted from collected cells and the abundance of individual sgRNAs is determined by high-throughput sequencing. Comparative analysis of sgRNA representation before and after selection enables the identification and ranking of functional genes that contribute to the phenotype under investigation.

The development of CRISPR-based screening technologies has enabled systematic interrogation of gene function at genome scale [52]. Pooled CRISPR knockout, activation, interference and precision editing screens, as well as combinatorial screens, allow identification of functional genes or genetic elements regulating diverse cellular phenotypes. For a typical pooled CRISPR screen, multiple (usually from 10^2^ to 10^5^) designed single-guide RNAs (sgRNAs) oligos are first synthesized in a chip and then collectively cloned into a lentiviral expression vector to construct a plasmid library. These sgRNA-expressing cassettes are introduced into targeted cells via lentiviral infection. The samples are collected before and after the infliction of different selection pressures associated with the process of interest by either direct collection of viable cells or flow cytometry-based sorting of target cells. Following genomic DNA extraction and PCR amplification of sgRNA cassettes, high-throughput sequencing is conducted for sgRNA quantification. Bioinformatic analysis of sgRNA read counts reveals differential enrichment or depletion across sample groups, enabling the identification of phenotype-driving genes [53] (Figure 2). Integration with single-cell sequencing technologies, such as Perturb-seq, CROP-seq and related multi-modal platforms, has further enhanced the investigation resolution by linking perturbations to transcriptomic outcomes at the single-cell level [54,55,56]. Such CRISPR-based genetic screens have found broad utility in numerous biological contexts, with prominent applications in oncology, metabolic disorders, and cardiovascular pathologies. In these settings, they demonstrate exceptional capacity for both unbiased discovery of critical genetic determinants and systematic elucidation of functional interactions that shape disease-related processes [57,58].

## 3. CRISPR Applications in T2D Research

While foundational applications of CRISPR technologies in T2D have been reviewed [19,20,59,60,61,62], emerging studies, especially those published from 2023 onward, largely remain undigested. Our present review aims to comprehensively synthesize these recent findings on this important topic.

### 3.1. CRISPR-Based Genetic Factor Modeling and Functional Analysis

**Figure 3:**
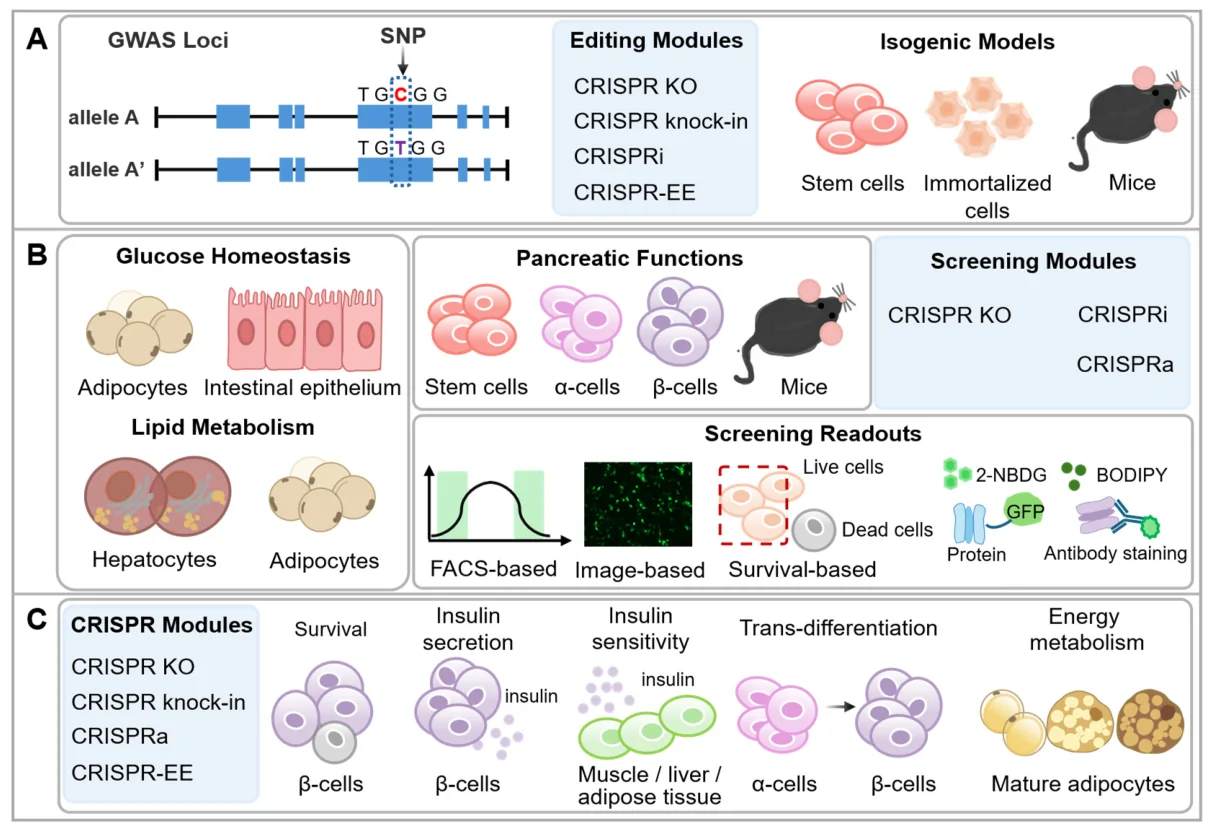
Schematic overview of CRISPR application in T2D research. (**A**) GWAS-associated loci and candidate SNPs can be modeled and functionally investigated using different CRISPR editing modules, including CRISPR KO, targeted knock-in, CRISPRi, and CRISPR-EE (epigenome engineering). (**B**) CRISPR-based screening can be applied in different cellular or animal models to identify functional genes underlying T2D-related processes, including glucose homeostasis, lipid metabolism and pancreatic functions. (**C**) CRISPR-based perturbation strategies can be deployed in therapeutic approaches against diabetes, such as restoration or enhancement of pancreatic cell functions, improvement of insulin sensitivity, and induction of *β*-cell regeneration via cellular reprogramming.

The genetic architecture of T2D is highly complex, with GWAS currently identifying over 600 genetic loci associated with disease risks [3]. However, approximately 90% of T2D-associated signals are located in non-coding genomic regions, and the biological functions and regulatory target genes of these regions remain largely unknown, posing a major bottleneck in translating genetic associations into causally functional mechanisms [63]. By combining CRISPR-Cas9 with HDR-based precise gene editing, researchers are able to introduce risk alleles into specific loci or correct them to protective alleles in hPSCs or immortalized cell lines, thus evaluating their direct impact on cellular phenotypes within an isogenic context [64,65,66] (Figure 3A).

One of the top T2D risk genes, *TCF7L2*, whose variations in its intronic region (single-nucleotide polymorphism SNP rs7903146) were associated with the disease according to GWAS results, has been extensively investigated [67]. In 2012, it was found that selective deletion of *TCF7L2* in the mice pancreas replicates key aspects of the altered glucose homeostasis in human carriers of *TCF7L2* risk allele [68]. The application of CRISPR further facilitated the generation of various genetic models. The endogenous *TCF7L2* locus in G456 mouse embryonic stem cell lines was targeted using CRISPR-Cas9 components (sgRNA and Cas9 mRNA) along with a loxP donor vector, yielding the *TCF7L2^Flox/Flox^* mouse lines [69]. To generate liver-specific *TCF7L2* knockout mice, *TCF7L2^Flox/Flox^* littermates were injected with AAV8-TBG-Cre. Subsequent analyses showed that liver-specific deletion of *TCF7L2* had minimal effects on plasma glucose levels but markedly disrupted hepatic zonation [70].

Circadian rhythm disruptions, modulated by melatonin, are linked to increased T2D risk, with the *MTNR1B* variant rs10830963 (G-allele) identified as a risk-associated expression quantitative trait locus (eQTL) in pancreatic islets, potentially impairing *β*-cell function [71,72]. Both the hiPSCs (human induced PSCs, reprogrammed from patient-derived fibroblasts) and the human embryonic stem cell (hESC) line HUES4 were independently subjected to single-base genome editing using CRISPR/Cas9 components (Cas9 protein Alt-R v3, custom sgRNAs, and ssDNA donors). This approach generated isogenic lines carrying either the non-risk C/C or the risk G/G genotype for each cell type. Elevated MTNR1B protein levels, increased sensitivity to melatonin and less insulin secretion were observed in stem cell-derived *β*-like cells carrying the risk G allele. This suggests that rs10830963 may contribute to *β*-cell dysfunction [73]. In addition, *MT1^−/−^* (M*TNR1A* KO) and *MT2^−/−^* (M*TNR1B* KO) mice were generated on a C57BL/6 background using a previously published method, in which Cas9 mRNA and sgRNA were co-injected into zygotes harvested from C57BL/6J mice [74,75]. CRISPR/Cas9-mediated knockout of *MTNR1A* and *MTNR1B* was used to investigate the role of melatonin signaling, revealing that loss of melatonin signaling impaired metabolic homeostasis and increased the risk of metabolic disorder–induced T2D [76].

Meta-analyses of GWAS in the European population have identified the *STARD10* locus as a T2D risk locus [77]. *β*-cell-specific *STARD10* KO mouse islets also showed a diminished cytosolic Ca^2+^ influx and insulin secretion in response to high glucose stimulation [78]. Similarly, deletion of *STARD10* in the human *β*-cell line EndoC-*β*H1 reduced glucose-stimulated insulin secretion [79]. In this study, researchers used CRISPR/Cas9 with sgRNA-mediated targeting to knockout *STARD10* in MEL1 INS^+^/GFP hESCs, successfully generating two *STARD10* knockout lines that were subsequently differentiated into *β*-like cells. The results provided further insight into the role of *STARD10* in human *β*-cell development and function, demonstrating how its loss may contribute to an increased risk of T2D [80].

*RGPR-p117* (also known as *SEC16B*) is an endoplasmic reticulum protein that has been shown to play a role in lipid export [81]. *SEC16B* is linked to T2D in a human GWAS [82], suggesting its role in regulating glucose homeostasis. *SEC16B* knockout mice were generated on a C57BL/6J genetic background using a CRISPR/Cas9-based approach. The study identified *SEC16B*, an obesity susceptibility gene previously identified through GWAS, as an evolutionarily conserved regulator of glucose homeostasis [83].

In addition to protein-coding genes, non-coding variants linked to T2D were also investigated via CRISPR knockout and CRISPRi perturbation modalities. For instance, the Huangfu group utilized a dCas9-KRAB-based CRISPRi screen in hPSC to identify enhancers functionally important for pancreatic development. They found that *ONECUT1e-664kb* enhancer deletion (via paired gRNA knockout) disrupts *ONECUT1* induction and hampers pancreatic differentiation, and the T2D non-coding variant rs528350911 in *ONECUT1e-664kb* (installed via CRISPR-based knockin using Cas9/sgRNA/ssDNA donor) decreases GATA4, GATA6, and FOXA2 binding [84]. A similar study was also performed for the *UBE2E2* locus where non-coding single nucleotide variants in cis-regulatory regions are associated with both T2D and visceral adiposity [85].

Epigenome editors, such as CRISPR-dCas9-DNMT3A or CRISPR-dCas9-TET1, were employed to install or erase DNA methylation of key T2D genes (e.g., *INS*, *TH* or *GCG*) in human EndoC-*β*H1 cells, respectively. This study revealed a causal link between DNA methylation and T2D gene regulation, and provided another perturbation angle to explore genetic causes of T2D risk factors [86].

### 3.2. CRISPR Screening Application in T2D Research

Beyond single-gene studies, genome-wide or high-throughput CRISPR screening has emerged as a powerful platform for systematically identifying and functionally characterizing genes involved in T2D pathogenesis (Figure 3B; Table 1).

#### 3.2.1. Application of CRISPR Screening in Glucose Homeostasis

Glucose metabolic dysregulation is a core pathophysiological hallmark of T2D, and its impacts are systemic and progressive, spanning the entire trajectory from disease onset to the development of complications. In T2D research, CRISPR screening has been used to systematically map the genetic factors that regulate glucose homeostasis under physiologically relevant metabolic stress.

To identify key regulators of glucose uptake, our group performed multiple fluorescence-activated cell sorting (FACS)-based genome-wide CRISPR loss-of-function screens in human SW872 preadipocyte-derived beige adipocytes targeting ~20,000 protein-coding genes. A fluorescent glucose analog (2-NBDG) was used to monitor glucose updateuptake and served as the FACS marker. After comparing the high- vs. low-signal groups, we identified a clethra of glucose metabolism regulators and validated *COMMD7* as a key gene in mediating glucose uptake under insulin-resistance (IR) conditions [87]. A similar strategy applied in murine brown preadipocytes identified the E3-ubiquitin ligase Rfwd2 a negative regulator of glucose uptake in brown adipocytes [88].

Gulbranson et al. developed a fluorescent reporter-based CRISPR screening platform to identify regulators of insulin-stimulated glucose transporter type 4 (GLUT4) exocytosis, a critical step for glucose uptake in adipocytes and skeletal muscles in response to insulin. The genome-wide CRISPR knockout screen was performed in HeLa cells and revealed *RABIF* as a positive regulator of GLUT4 exocytosis by stabilizing Rab10 [89]. The Lippmann group performed an arrayed CRISPR knockout screens targeting more than 8000 genes in human Caco-2 cells to identify regulators of GLUT1 expression, another glucose transporter. They discovered more than 300 genes whose loss-of-function led to GLUT1 downregulation and many of these genes were classified as G-protein coupled receptors [90].

#### 3.2.2. Application of CRISPR Screening in Lipid Metabolism Related to T2D

Lipid metabolic dysregulation is a core pathophysiological hallmark of T2D, with systemic and progressive effects that extend from disease onset to the development of complications.

The beiging of white adipocytes enhances energy expenditure by increasing the utilization of fatty acids and glucose, thereby offering therapeutic potential for obesity and T2D. To identify key regulators of beige adipocyte function, our group employed FACS-based genome-wide CRISPR KO screens in human SW872 preadipocytes and identified functional genes regulating beige adipocyte differentiation. Lipid droplet stainer BODIPY dye and Side Scatter (SSC) were used to assess adipogenic capacity in flow cytometry analysis. These screenings identified *SULT2B*1 and *ATP1B2* as key regulators of beige adipogenesis [87].

Olzmann and colleagues employed a genome-wide human CRISPR knockout library (including 212,821 sgRNAs targeting 20,549 genes) as well as a custom Validation of Lipid Droplet and Metabolism (VLDM) sgRNA library (including 8550 sgRNAs targeting 857 genes) to explore lipid storage and secretion in human hepatocytes (Huh7 hepatoma cells). BODIPY 493/503 fluorescence served as the FACS reporter. They pinpointed *CLCC1* as a critical regulator of neutral lipid storage and secretion, whose loss-of-function led to the buildup of large lipid droplets in hepatocytes and caused liver steatosis in mice [91]. The role of *CLCC1* to maintain lipid homeostasis was also recapitulated by another back-to-back study led by Chen et al. [92].

Turn et al. performed a FACS-based genome-wide CRISPR knockout screen in mouse 3T3-L1 preadipocyte cells using an sgRNA library comprising approximately 10 sgRNAs per gene targeting ~20,500 protein-coding genes [93]. The lipophilic fluorescent BODIPY dye served as the monitor of lipid content and FACS marker. They found that, specific branches of the hypusination pathway, a conserved regulator of translation initiation, are critical for adipogenesis, and the neddylation/ubiquitin pathway modulates insulin sensitivity during lipogenesis [94].

To investigate the functions of small open reading frames (smORFs) during adipocyte proliferation and differentiation, Pai et al. performed a CRISPR dropout screen targeting 1737 unannotated adipocyte-smORFs in murine 3T3-L1 adipocytes with FACS-sorted lipid droplets as a readout. They identified dozens of smORFs that regulate adipocyte proliferation and lipid metabolism [95].

An array-form CRISPR knockout screen using imaging-based morphologic profiling (e.g., BODIPY staining) was performed to functionally perturb 125 T2D relevant genes according to human genetics in human Simpson–Golabi–Behmel Syndrome (SGBS) preadipocyte cells. The authors identified 14 genes whose knockout decreases lipid accumulation and validated two pairwise gene interactions (*BSCL2* and *PLIN1*; *CEBPA* and *AGPAT2*) for further mechanistic insights [96].

A CRISPRi-based (dCas9-KRAB) screen was employed to map the candidate cis-regulatory regions for the *UBE2E2* gene, a regulator of adipogenesis, in immortalized adipose-derived human mesenchymal stem cells (hMSCs). The expression level of *UBE2E2* was used as the FACS marker. This screen uncovered key cis-regulators of *UBE2E2* and functional non-coding variants for T2D [85].

For CRISPR screens in other aspects of lipid metabolism, such as cholesterol homeostasis and cardiovascular diseases, this is beyond the scope of the present review and readers might refer to other references [53,57].

#### 3.2.3. Application of CRISPR Screening in Pancreatic Functions

Pancreatic dysfunction is central to the pathophysiology of diabetes, characterized by abnormal insulin secretion pulses, dedifferentiation into non-functional endocrine cells, and ultimately programmed cell death [97]. CRISPR technology has made it possible to systematically dissect the molecular determinants of maintaining pancreatic cell identity and function at the genomic level [98].

Glucagon, secreted by pancreatic *α*-cells, is essential for maintaining glucose homeostasis. In type 1 diabetes and advanced T2D, *α*-cells often fail to respond appropriately to hypoglycemia. To identify regulators of *α*-cell function, researchers performed a genome-wide CRISPR screen in AlphaTC6 cells using the mouse Brie pooled CRISPR knockout library, which targets 19,674 genes with four sgRNAs per gene. The screen identified *SEC31A*, a gene involved in endoplasmic reticulum (ER)-to-Golgi protein transport, as a key regulator of *α*-cell survival under stress [99].

Pancreatic *β*-cells are the primary insulin-producing cells in the body and *β*-cell dysfunction underlies the development of diabetes. Okino et al. performed a loss-of-function CRISPR screen in EndoC-*β*H1 cells using the human Brunello library (targets 19,114 genes with 76,441 sgRNAs) to identify regulators of *β*-cell survival under ER stress. The screen preliminarily identified 167 pro-survival genes and 47 pro-death genes, including the novel T2D candidate gene *DTNB*, which was validated to protect *β*-cells against stress-induced cell death [100]. A CRISPRi (dCas9-KRAB) screen was also performed to explore putative enhancers for pancreatic development (particularly *β*-cell lineage specification) using hPSC differentiation model and PDX1 expression as the FACS marker. A long-range enhancer  ~664 kb away from the *ONECUT1* promoter was pinpointed to regulate pancreatic differentiation [84]. Li et al. carried out a genome-scale CRISPR knockout screen (GeCKO v2 library with ~120,000 sgRNAs targeting 19,050 genes) to identify key genes mediating human pancreatic duct cell trans-differentiation into functional *β*-like cells using pancreatic REPB-PANC-1 fluorescence reporter cell line. This work showed that loss-of-function of *ALDH3B2* is sufficient to trans-differentiate human pancreatic duct cells into functional *β*-like cells [101].

Altered proinsulin levels in *β*-cells and bloodstream are hallmarks of diabetes. Lai et al. carried out a genome-wide CRISPR screen in a mouse *β*-cell line (MIN6) to determine proinsulin regulators by monitoring FACS-based intracellular proinsulin/insulin ratio. They identified 84 proinsulin regulators, and validated *Pdia6* as the strongest hit whose loss-of-function significantly reduces proinsulin accumulation in Golgi and secretory granules [102]. The same group also employed a similar CRISPR screen and experimental system (using insulin expression level as a FACS marker), and determined the NuA4/Tip60 histone acetyltransferase complex as a critical insulin regulator among others [103]. Human pancreatic *β*-cell line EndoC-*β*H1 and the TKOv3 CRISPR library (targeting 18,053 protein-coding human genes with four sgRNAs per gene) were also used for FACS-based genome-wide loss-of-function screen in search of genetic regulators underlying intracellular insulin content. This work identified 580 genes influencing this phenotype and validated the autophagy receptor *CALCOCO2* as a prominent insulin regulator [98]. Another study employed single-cell RNA sequencing to analyze genome-wide lentiviral CRISPR knockout library-edited mouse MIN6 cells for studying insulin transcription. The lncRNAs *GM26917* and *Cenpw* were found to be associated with insulin transcription [104]. Single-cell RNA sequencing on pooled CRISPR screens (Perturb-seq using Zim3 KRAB-dCas9 CRISPRi system) was also applied to EndoC-*β*H1 cells using a targeted sgRNA library comprising 61 T2D-associated genes and 40 ribosome-associated quality control (RQC) genes. This screen systematically characterized the functions of these candidate genes in insulin production and T2D pathogenesis. The analysis identified 21 functional genes, including the previously uncharacterized *KLHL42* and *ZZEF1*. Further functional validation demonstrated that *ZZEF1* is a regulator of insulin synthesis and *β*-cell stress through ribosomal stress-surveillance pathways [105]. To identify the genetic regulators for insulin secretion, Yang et al. deployed a genome-wide CRISPR knockout screen (containing 78,637 sgRNAs targeting 19,674 genes) in a MIN6 reporter line (labeling exocytic insulin granules). This study identified several members of the COMMD family, with central roles in intracellular membrane trafficking, as positive regulators of basal insulin secretion via ITGB1 recycling [106]. Another work utilized a focused CRISPR knockout library targeting 83 K^+^ channels with 332 sgRNAs in MIN6 cells and proceeded to single-cell RNA sequencing analysis. The authors found that Kcnb1 and Kcnh6 were the two most important repolarization K^+^ channels for high-glucose-dependent insulin secretion [107]. Regulators for insulin signaling was investigated through a CRISPRi (dCas9-KRAB) screen in HeLa cells using a focused mitochondria-related gene library containing 12,300 sgRNAs. The FOXO1 subcellular localization served as a phenotypic readout for FACS. This work identified 117 candidate regulators and prioritized *NAA25* as a key regulator of FOXO1-mediated insulin signaling [108].

Genetic engineering of hPSC-derived islets represents a promising strategy for improving islet transplantation in diabetes cell therapy. In 2022, researchers employed an in vivo genome-wide CRISPR screen to identify genetic perturbations that reduce immune rejection of stem cell-derived islets (SC-islets) [109]. Human SC-islets edited by the Brunello CRISPR knockout library (76,441 sgRNAs targeting 19,114 genes) were transplanted to the NSG-MHC^null^ mouse model and selected against allogeneic human peripheral blood mononuclear cell (PBMC) injection. This screen confirmed that targeting IFNγ-induced mediators has beneficial effects on SC-islet survival under immune attack [109]. A recent study also probed this question using a genome-wide CRISPRa-based (dCas9-VPR) screen. HUES8-VPR stem cells were transduced with a lentiviral CRISPRa library (comprising 106,400 sgRNAs targeting 18,915 genes) and 3755 non-targeting control sgRNAs. After differentiation into SC-islets, these cells were transplanted into the kidney capsule of immunodeficient NSG mice to allow grafting. This screen identified multiple candidate genes, including *FCAMR* (Fc alpha/mu receptor), to be beneficial for stem cell-derived islet transplantation and glucose control [110]. Another study investigated the genetic protectors of autoimmune destruction of pancreatic *β*-cells using an in vivo genome-scale CRISPR knockout screen using the mouse lentiviral GeCKO library (~60,000 sgRNAs targeting ~19,050 genes). The library-edited murine NIT-1 *β*-cells were transplanted subcutaneously into immunodeficient NOD.scid mice and selected, and splenocytes from diabetic NOD mice were injected into transplant recipients to elicit *β*-cell killing. This screen identified *RNLS* as a target for *β*-cell protection [111].

**Table 1:** Representative functional genes identified by CRISPR screens in T2D.

Biological Process	Gene	Perturbation	Library	Model	Function	Reference
Glucose Homeostasis	*COMMD7*	Knockout	Genome-wide	SW872	*COMMD7* loss promoted glucose uptake under IR conditions	[87]
	*Rfwd2*	Knockout	Genome-wide	Brown preadipocytes	*Rfwd2* loss promoted glucose uptake in basal and insulin-stimulated state	[88]
	*RABIF*	Knockout	Genome-wide	HeLa	*RABIF* loss impaired insulin-stimulated GLUT4 exocytosis	[89]
	*YBX1*, *FBXL5*, and *USP47*	Knockout	Focused library	Caco-2	*YBX1*, *FBXL5*, and *USP47* depletion downregulated GLUT1 protein expression	[90]
Lipid Metabolism	*SULT2B1* and *ATP1B2*	Knockout	Genome-wide	SW872	*SULT2B1* or *ATP1B* depletion promoted beige adipocyte differentiation	[87]
	*CLCC1*	Knockout	Genome-wide; Focused VLDM library	Huh7	*CLCC1* loss led to the buildup of large lipid droplets in hepatocytes	[91,92]
	*NEDD8* and *DHPS*	Knockout	Genome-wide	3T3-L1	Loss of *NEDD8* promoted lipid accumulation; loss of *DHPS* inhibited adipogenesis	[94]
	*smORF-340, smORF-543* and *smORF-1183*	Knockout	Specialized adipocyte-smORFs	3T3-L1	*smORF-340* or *smORF-543* deletion inhibited adipocyte proliferation; *smORF-1183* deletion reduced lipid drop formation	[95]
	*BSCL2* and *PLIN1; CEBPA* and *AGPAT2*	Knockout	Metabolic disease gene library	SGBS adipocytes	Affected lipid accumulation and lipid droplet morphology	[96]
	*rs7374732*, *rs9812056*, *rs6780569*, *rs6792370*, *rs7612463*,	CRISPRi	Specialized sgRNA library targeting a single genomic locus	hMSCs	Affect the expression of *UBE2E2*, adiposity and glucose homeostasis	[85]
Pancreatic Functions	*SEC31A*	Knockout	Genome-wide	AlphaTC6	Lack of *SEC31A* protected *α*-cells from ER stress-induced cell death	[99]
	*DTNB*	Knockout	Genome-wide	EndoC-*β*H1	*DTNB* promoted *β*-cell survival in ER stress	[100]
	*ONECUT1e-664kb*	CRISPRi	Specialized library targeting enhancers	hPSCs	*ONECUT1-664kb* enhancer deletion resulted in impaired pancreatic differentiation	[84]
	*ALDH3B2*	Knockout	Genome-wide	PANC-1 cells	*ALDH3B2* loss increased trans-differentiation of pancreatic duct cells into *β*-like cells and insulin secretion	[101]
	*Pdia6*	Knockout	Genome-wide	MIN6	*Pdia6* loss reduced proinsulin accumulation in Golgi and secretory granules	[102]
	*NuA4/Tip60*	Knockout	Genome-wide	MIN6	NuA4/Tip60 HAT Complex regulated insulin secretion	
	*CALCOCO2*	Knockout	Genome-wide	EndoC-*β*H1	Silencing of *CALCOCO2* reduced insulin secretion	[98]
	*GM26917* and *Cenpw*	Knockout	Genome-wide	MIN6	Inhibition *GM26917* or *Cenpw* downregulated insulin level	[104]
	*ZZEF1*	CRISPRi	61 T2D-associated genes and 40 RQC genes	EndoC-*β*H1	*ZZEF1* deficiency impaired *β*-cell function	[105]
Pancreatic Functions	*COMMD3*	Knockout	Genome-wide	MIN6	*COMMD3* loss decreased basal insulin secretion in low glucose conditions	[106]
	*Kcnb1* and *Kcnh6*	Knockout	K^+^ channels	MIN6	Blocking *Kcnb1* or *Kcnh6* promoted insulin secretion	[107]
	*NAA25*	CRISPRi	Mitochondria-related gene library	HeLa	*NAA25* knockdown attenuated FOXO1-mediated insulin signaling	[108]
	*CXCL10*	Knockout	Genome-wide	SC-islets	Depleting *CXCL10* reduced SC-islet immunogenicity	[109]
	*FCAMR*	CRISPRa	Genome-wide	HUES8-VPR stem cells	*FCAMR* was beneficial for stem cell-derived islet transplantation and glucose control	[110]
	*RNLS*	Knockout	Genome-wide	Mouse	*RNLS* mutation protected *β*-cells from autoimmune killing	[111]

#### 3.2.4. Comparative Synthesis of CRISPR Screening in T2D Research

CRISPR modality fundamentally shapes screening outcomes. KO induces permanent, irreversible gene inactivation and strong loss-of-function effects, but may cause lethality, gene compensation, or clonal selection, especially for essential genes. CRISPRi provides reversible, titratable, dose-dependent knockdown that is better suited to essential and dosage-sensitive genes, although incomplete repression and chromatin or sgRNA context can cause false negatives. CRISPRa is useful for discovering gain-of-function targets, non-coding regulatory elements, and pathway activation, but is confounded by overexpression artifacts, toxicity, saturation, and context dependence. Consequently, the same gene may be detected in one screen but missed in another [112,113]. In T2D-relevant models, comparative analysis across KO, CRISPRi, and CRISPRa, combined with orthogonal validation, is helpful to pinpoint bona fide targets or functional hits.

The choice between genome-wide and focused CRISPR libraries involves a trade-off between resources, sensitivity and interpretability [114]. Genome-wide libraries enable unbiased discovery but require 500–1000-fold coverage and tens to hundreds of millions of cells, limiting their use in primary and differentiated models [115]. Focused libraries contain fewer guides, and usually enable higher sequencing depth and lower noise, but are hypothesis-driven and may miss novel T2D effectors outside predefined sets. Genome-wide screens are more prone to false positives and negatives from guide representation bias or uneven sgRNA uniformity [116]. Preliminary evidence from a preprint suggests that focused libraries can uncover false negatives and subtle effects missed by larger libraries [117]. Statistically, genome-wide screens dilute reads across many guides and lose precision and recall under low coverage, whereas focused libraries concentrate power on coherent gene sets and yield validation-ready shortlists [118]. Thus, genome-wide libraries suit unbiased discovery, whereas focused libraries suit hypothesis-bounded questions. For T2D research, a tiered strategy of genome-wide discovery followed by focused validation in disease-relevant models may best balance breadth and depth.

The cellular model, species, and phenotypic readout collectively determine which genes emerge as screening hits, making cross-study comparison essential yet challenging. Immortalized *β*-cell lines (e.g., EndoC-*β*H1, MIN6, and INS-1) are scalable but poorly recapitulate GSIS and islet interactions [119,120]. Primary human islets are more physiological but scarce and variable [121,122]. iPSC-derived *β*-cells enable human genetics but remain functionally immature [123]. Hepatocytes, adipocytes, and myotubes model distinct T2D axes—hepatic glucose output, adipose insulin sensitivity, and muscle insulin responsiveness—and can yield different hits [124]. Species differences further limit translation, as rodent findings are not always recapitulated in humans [98,125]. The readout is equally decisive: proliferation-based screens enrich essential genes, whereas insulin-content screens identify trafficking, autophagy, and secretory pathway genes [126]. Consequently, the same gene may be detected in one model but missed in another, making cross-model validation essential to distinguish context-dependent from broadly acting T2D regulators.

A major caveat in T2D CRISPR screening is that independent functional validation of hits varies widely. Validation ranges from individual sgRNA confirmation and orthogonal perturbation (e.g., RNAi, CRISPRi/a) to testing in independent models or species and, ultimately, integration with human genetics or in vivo studies [127]. *CALCOCO2* exemplifies a deep validation: it was identified in a genome-wide screen in EndoC-*β*H1, confirmed by shRNA knockdown in primary human pseudoislets, and supported by human T2D variant associations with insulin secretion [98]. Similarly, *DTNB* was identified in a CRISPR screen for *β*-cell survival under ER stress and independently validated to protect against *β*-cell death during stress, with the gene set showing significant enrichment for T2D risk variants [100]. In contrast, many hits from single cell line screens with one phenotypic readout remain unvalidated. Therefore, screening hits should be interpreted according to validation depth. Cross-model, orthogonal validation is needed to distinguish robust T2D effectors from preliminary candidates.

### 3.3. CRISPR-Guided Therapeutics Development for Diabetes

CRISPR technologies have enabled gene-based therapeutic strategies that offer precise approaches for targeting the molecular mechanisms underlying diabetes. A major focus of CRISPR-guided therapeutics is the restoration of pancreatic *β*-cell function through correction of pathogenic genetic variants, protection against stress-induced *β*-cell damage, improvement of insulin sensitivity, and induction of *β*-cell regeneration via cellular reprogramming from hPSCs or *α*-cells [62,128] (Figure 3C). CRISPR/Cas9 has demonstrated considerable potential for in vitro or ex vivo genome editing in embryonic stem cells and patient-derived stem cells, facilitating investigations into *β*-cell development and function, the generation of novel diabetic models, and the evaluation of therapeutic approaches [62,129].

Millman and colleagues employed a CRISPR/Cas9-mediated HDR-dependent gene correction approach to repair a diabetes-causing pathogenic variant of *WFS1* gene in patient-derived hiPSCs. After in vitro differentiation, the edited hiPSC-derived *β*-cells were transplanted into diabetic mice, which showed robust dynamic insulin secretion in response to glucose and reversed pre-existing streptozocin-induced diabetes [130].

DPP-4 is responsible for rapid degradation of incretin hormones such as GLP-1, and several DPP-4 inhibitors have been developed for the treatment of T2D [131]. Cho et al. developed a lecithin-based liposomal nanocarrier particle to encapsulate DPP-4-targeting Cas9/sgRNA ribonucleoprotein, and targeted disruption of *DPP-4* was achieved in T2D *db*/*db* mice (especially in liver) via intravenous injection. This approach successfully reversed diabetes phenotypes in treated mice, providing a proof-of-concept for in vivo gene therapy against T2D [132].

NRIP1 strongly suppresses glucose transport, fatty acid oxidation, and mitochondrial respiration, serving as an attractive target to enhance the therapeutic potential of adipocytes in obesity and diabetes. Tsagkaraki et al. knocked out *NRIP1* in mouse and human adipocyte progenitor cells ex vivo using Cas9/sgRNA ribonucleoproteins to switch towards brown adipocytes, which display enhanced glucose and fatty acid utilization as well as heat production. Implantation of these CRISPR-enhanced cells into high-fat-diet-fed mice decreases adiposity and liver triglycerides while enhancing glucose tolerance [133].

Beyond its conventional application in gene disruption, CRISPR-based technologies can also be harnessed to modulate *β*-cell fate and function, providing opportunities for protecting *β*-cells from metabolic stress and promoting regenerative processes [134,135,136]. The CRISPRa system provides a promising framework for this strategy by fusing dCas9 with transcriptional activators, enabling targeted upregulation of endogenous gene expression without modifying the underlying DNA sequence. CRISPRa can potentially enhance the expression of key regulatory genes involved in *β*-cell regeneration and insulin sensitivity, including *PDX1*, *MAFA*, NGN3, *IRS2*, and *FOXO1* [137]. Although this approach remains largely theoretical and requires extensive experimental validation, CRISPRa-based gene activation represents a promising therapeutic strategy for future precision medicine approaches in T2D.

*TXNIP* plays a critical role in T2D pathogenesis: it is upregulated under hyperglycemic conditions, promotes *β*-cell apoptosis and oxidative stress, and is considered a key mediator of pancreatic *β*-cell dysfunction and failure [138]. CRISPR-dCas9-mediated CpG island editing represents an emerging strategy for T2D therapeutics via targeted epigenetic reprogramming of *TXNIP* expression, offering potential therapeutic benefits for diabetes management [136]. In parallel, the researchers used the modular epigenetic CRISPR/dCas9 toolbox for targeted DNA methylation (EpiCRISPR) and silencing of the *ARX* gene. Methylation-based silencing of *ARX* initiates the reprogramming of pancreatic *α*-cells into insulin-producing cells. As a key novelty, this protocol provides a direct route for epigenetically induced trans-differentiation of mouse pancreatic alpha TC1-6 cells into insulin-producing cells and thereby confirms a proof-of-concept of reversible cellular epigenetic reprogramming in vitro [139].

Although encouraging basic and pre-clinical results have been obtained, these progresses are largely experimental proof-of-concept and CRISPR-involved cell and gene therapies against T2D remain undeployed in real clinical settings. The prospect of a one-shot cure for T2D in humans remains speculative and must await rigorous clinical validation beyond the current pre-clinical observations. The safety concern, delivery bottleneck, and high cost collectively limit the application of CRISPR-based therapeutics in T2D. In addition to serving as direct therapeutics, CRISPR technology has always been helpful in identifying the genetic targets of therapeutic value and deciphering mechanism-of-action for therapeutic agents as stated in previous sections.

## 4. Challenges of the CRISPR Technology Applications in T2D

Although CRISPR technology holds great promise, its application in T2D research is still constrained by several key technical bottlenecks.

### 4.1. Limitations of Cell Models

One critical yet often overlooked factor is the limitations of the cell models on which CRISPR technology relies. Most applications of CRISPR technology are conducted in immortalized tumor cell lines (e.g., HeLa, Huh7, and HepG2) or a few immortalized human pancreatic *β*-cell models (e.g., EndoC-*β*H1/H3). While these cell lines have the advantages of strong proliferative capacity and ease of culture, their differences from primary human cells severely affect the physiological relevance, genetic diversity, cellular heterogeneity and clinical translatability of experimental results [140,141,142].

### 4.2. Editing Efficiency

In addition to the limitations of cell models, editing efficiency is still low in fully differentiated primary cells (e.g., human pancreatic *β*-cells) for precision editing. Although the NHEJ-dependent indel generation by Cas9 nuclease is highly efficient, precise HDR-based editing strategies are often difficult to implement, mainly due to the relatively quiescent cell cycle status and limited DNA repair mechanisms in these cells [122]. Although base editors are capable of robust editing activity, their performance is highly contingent on the chosen endogenous target sites. The editing efficiency of prime editors is still generally low, awaiting further optimization.

### 4.3. Delivery Systems

CRISPR/Cas9 technologies are delivered through several methods, including viral (e.g., lentivirus and adeno-associated virus) and non-viral approaches (e.g., lipid nanoparticles). These systems have different drawbacks and risks, such as off-target effects, host genome integration, unwanted immune responses, limited packaging capacity, delivery efficiency, in vivo targeting specificity and stability [143,144,145]. These concerns should be considered when applying CRISPR modules for both basic research and therapeutic scenarios.

### 4.4. Safety and Ethics

For CRISPR-based cell and gene therapies, the long-term safety profiles in humans need to be carefully addressed on the path to broad clinical application. From an ethical and regulatory perspective, finding a balance between promoting innovation and ensuring safety and fairness remains a topic for discussion across all sectors of society [145,146,147].

## 5. Conclusions

The application of genome-wide CRISPR screening technology signifies a new stage in our shift from studying individual gene functions to systematically analyzing complex gene networks in the field of T2D research. Using CRISPR-based approaches, scientists have not only validated the functional importance of hundreds of T2D risk loci identified through GWAS, but also discovered new regulatory factors. These findings have unveiled previously unknown molecular pathways involved in insulin secretion, *β*-cell survival, glucose and lipid metabolism. These discoveries not only bridge the knowledge gap between genetic associations and biological mechanisms, but also provide a molecular basis for understanding the heterogeneity in T2D pathogenesis.

Looking forward, the deployment of CRISPR technology in T2D research will show several key trends: First, novel editing tools, such as base editing and prime editing, will provide safer and more precise genomic modifications to better resolve the genetic underpinnings. Second, multi-omics integration strategies will reveal dynamic changes in gene regulatory networks at the temporospatial and single-cell levels. Third, more advanced in vitro models (such as organ-on-a-chip and organoids) will provide higher throughput and more physiologically relevant screening platforms compared to simple cell culture. Finally, personalized treatment strategies may achieve true precision medicine by genetics-informed cell and gene therapies. These efforts will ultimately attain a qualitative leap from symptom management to disease modification for T2D patients.

## Data Availability

No new data were created or analyzed in this study. Data sharing is not applicable to this article.

## Abbreviations

The following abbreviations are used in this manuscript:

T2D	Type 2 diabetes
GWAS	Genome-wide association studies
CRISPR	Clustered regularly interspaced short palindromic repeats
GLP-1 RAs	Glucagon-like peptide 1 receptor agonists
SGLT2i	Sodium-Glucose co-transporter 2 inhibitors
KO	knockout
CRISPRi	CRISPR interference
CRISPRa	CRISPR activation
CRISPR-EE	CRISPR epigenome engineering
RNAi	RNA interference
hPSCs	Human pluripotent stem cells
SpCas9	Streptococcus pyogenes Cas9
DSBs	Double-strand breaks
NHEJ	Non-homologous end joining
HDR	Homology-directed repair
dCas9	Catalytically dead Cas9
ABEs	Adenine base editors
CBEs	Cytosine base editors
nCas9	Cas9 nickase
pegRNA	Prime editing guide RNA
sgRNA	Single guide RNA
SNP	Single nucleotide polymorphism
eQTL	Expression quantitative trait locus
hESC	Human embryonic stem cell
FACS	Fluorescence-activated cell sorting
IR	Insulin resistance
GLUT4	Glucose transporter type 4
smORFs	Small open reading frames
SGBS	Simpson–Golabi–Behmel syndrome
hMSCs	Human mesenchymal stem cells
ER	Endoplasmic reticulum
SC-islets	Stem cell-derived islets
